# Book Reviews

**Published:** 1895-01

**Authors:** 


### *BOOK REVIEWS.*

A Dictionary of Medicine. By various writers. Edited by Richard Quain, M.D., LL.D., F.R.S., London; assisted by Frederick T. Roberts, M.D., and J. Mitchell Bruce, M.D., London, with an American appendix by Dr. Samuel T. Armstrong, New York. New edition, revised and enlarged. Two volumes. D. Appleton & Co., New York.

The motive which produced this original work in 1882, was to "place in the hands of the practitioner, the teacher and the student a means of ready reference to the accumulated knowledge which we possess of scientific and practical medicine." It endeavored to present "all the information available for the use of the practitioner." The success attained by the first edition and the progress made in medicine within the intervening twelve years have demanded a new edition, enlarged and thoroughly revised up to date. The work is now issued in two volumes, containing 700 pages more than the one-volume edition, and the number of illustrations has also been increased.

The subjects are arranged in alphabetical order, thus providing for easy reference. The contributors have been selected (chiefly from English authorities) from their peculiar qualifications for the subjects described by them. The description of diseases is thorough and complete, every allowance being made for late advances in pathology, and therapeutics. Each paper is thus a monograph from an acknowledged authority, and the sum total is almost a library in itself.

While the work is primarily a dictionary of medicine, certain diseases ordinarily classed as surgical receive full consideration; as, pyemia, tubercle, syphilis, gonorrhea, tumors, etc. Also, the general principles of hygiene, sanitation, disinfection, quarantine, and vital statistics are well presented in their most practical relations. The American appendix by Dr. Armstrong increases the value of the book for American readers.

As a useful, practical reference-book of medicine, Quain's Dictionary must be regarded as a standard of the highest value. We cordially recommend, indeed advise, our readers to get it. It will well repay you.

---

SYLLABUS OF LECTURES ON HUMAN EMBRYOLOGY: WITH A GLOSSARY ON EMBRYOLOGICAL TERMS. By Walter Porter Manton, M.D., Professor of Clinical Gynecology and Lecturer on Obstetrics in the Detroit College of Medicine, etc. Illustrated with seventy Outline Drawings and Photo-Engravings. 12 mo. Cloth, 126 pages, $1.25 net. Philadelphia: the F. A. Davis Co., Publishers, 1914 and 1916 Cherry Street.

"The object of this syllabus is to furnish to students of medicine and practitioners an outline of the principal facts of human embryology." Details and theories are omitted. The author's purpose is excellently carried out, his syllabus being a very good presentation of the facts in human embryology and development. It forms an excellent introduction to the study of obstetrics and gynecology, much better and more complete than corresponding portions of text-books on these subjects. The illustrations are very good, and greatly assist the understanding of this somewhat difficult subject. We cheerfully commend this little work on embryology.

---

MANUAL OF MODERN SURGERY. By John C. DaCosta, M.D., Demonstrator of Surgery, Jefferson Medical College, Philadelphia, etc. $2.50. W. B. Saunders, Publisher, 925 Walnut Street, Philadelphia.

In this work the author attempts to present the golden mean between the "complete but cumbrous text-book and the incomplete but concentrated compend." He has succeeded with his task very well giving the reader a condensed and practical manual of modern surgery, not without its faults, it is true, but with its merits also; among the latter is the fact that it is a modern work, representing modern surgical principles and practices. It is rather loosely written; the author's rhetoric is faulty and his grammar badly out of joint in many places. For instance, we are told (p. 668) "for people over sixty . . . . ether fills them up with mucus," etc. Many such examples of bad English can be found, which cannot but injure the value of the work. The author's teachings seem to accord with modern ideas. He has tried not to depart too far from conservative methods, at the same time making proper mention of late recognized advances. This is especially true of abdominal and cranial surgery. However, it would hardly be considered conservative or wise to shoot off a finger or two with a pistol in case of snake-bite of these members (p. 159). This work will find its greatest usefulness as a manual for students; to this extent we take pleasure in commending it with all its loose constructions and other evidences of hasty compilation. On the whole, we think students will find this a very serviceable and convenient manual of modern surgery.

---

ESSENTIALS OF DISEASES OF THE SKIN, INCLUDING THE SYPHILODERMATA, arranged in the form of questions and answers, prepared especially for Students of Medicine. By Henry W. Stelwagow, M.D.; Ph.D., Clinical Professor of Dermatology in Jefferson Medical College, etc., etc., etc. Philadelphia: W. B. Saunders, 1894. $1.00.

This is the third edition, revised and enlarged, having seventy-one letter-press cuts and fifteen half-tone illustrations. The anatomy of the skin is given by cuts with the description beneath them simply. The common arrangement of works on dermatology is followed. The text is brief but clear. The little book contains many practical points, so arranged as to be useful not only to the beginner, but to the dermatologist. The illustrations, mostly from other works, are very good. M. B. H.

---

PHYSICIAN'S VISITING LIST FOR 1895. P. Blakiston Son & Co., 1012 Walnut street, Philadelphia. Price, $1.00.

This visiting list is one of the most popular in use, having reached the forty-fourth year of publication. It is conveniently arranged for twenty-five patients per week, and contains pages for general memoranda, obstetric records, cash account, etc. At the beginning are the usual tables on Examination of Urine, New Remedies, Calculating the Period of Gestation, etc., which will be found useful.

A HAND-BOOK OF MEDICAL MICROSCOPY FOR STUDENTS AND GENERAL PRACTITIONERS, INCLUDING CHAPTERS ON BACTERIOLOGY, NEOPLASMS, AND URINARY EXAMINATIONS. By James E. Reeves, M.D., Member of Association of American Physicians, etc., etc., etc. Philadelphia: P. Blakiston Son & Company.

This is a very complete, thorough, and practical little book, by an author whose long experience and excellent work well fit him for its production. A beginner or a microscopist of experience will find it of great value to him. An especial feature is the collection of various subjects in one volume, thus rendering it not necessary to consult many books for aid in microscopy. M. B. H.

---

MEDICAL REVIEW VISITING LIST. Perpetual. Price $1.00. J. H. Chambers & Co., St. Louis, Mo.

This is a perpetual visiting list, arranged for twenty-five patients per week. It contains also the usual pages for other memoranda, and tables of reference. Physicians will find it to answer all the purposes of a good visiting list.

---

LIPPINCOTT'S MAGAZINE FOR JANUARY, 1895.

The complete novel in the January issue of *Lippincott's* is "The Waifs of Fighting Rocks," by Captain Charles McIlvaine. The scene is laid in the mountains of West Virginia, and the tale is one of adventure, love, and jealousy among the mountaineers.

"By Telephone," a stirring newspaper story by Francis C. Regal, shows how a plucky reporter defeated a conspiracy and brought the criminals to justice. "A Question of Responsibility," by Imogen Clark, deals with delicacy *vs.* life-saving in a lodging-house.

The other stories belong to Christmas, and are offered at the right time instead of a month beforehand, as is the usual magazine custom. These are "Mrs. Santa Claus," by Marjorie Richardson, "A Prodigal Friend," by S. Elgar Benet, and "Mrs. Risley's Christmas Dinner," by Ella Higginson. Each of them is in the spirit of the season, though the last is in a minor key.

"Christmas Customs and Superstitions" are collected by Elizabeth Ferguson Seat. Edgar Fawcett recalls "New Year's Days in

Old New York," and Edith Duff "Empress Josephine's Happy Day," ninety years ago.

In "The Ducks of the Chesapeake" Calvin Dill Wilson tells all about the canvas-back before he is shot and after. Gilbert Parker offers a study of "Herbert Beerbohm Tree," the actor. F. M. B., in "With the Autocrat," recalls some notable private utterances of Dr. Holmes, and M. Kaufmann discusses "Socialist Novels."

The poetry of the number is by M. S. Paden, Alice Brown, Kathleen R. Wheeler, and Susie M. Best.

---

### THE INTERNATIONAL MEDICAL ANNUAL.

E. B. Treat, publisher, New York, has in press for early publication the 1895 International Medical Annual, being the thirteenth yearly issue of this eminently useful work. Since the first issue of this one volume reference work, each year has witnessed marked improvements; and the prospectus of the forthcoming volume gives promise that it will surpass any of its predecessors. It will be the conjoint authorship of thirty-eight distinguished contributors and specialists, from America, England and the continent. It will contain the progress of Medical Science in all parts of the world, together with a large number of original articles and reviews by authors on subjects with which their scientific reputation is identified. In short, the design of the book is to bring the practitioner into direct communication with those who are advancing the Science of Medicine, so he may be furnished with all that is worthy of preservation, as reliable aids in his daily work. Illustrations in black and colors will be freely used in elucidating the text. A most useful investment for the medical practitioner. The price remains the same as heretofore, $2.75.

